# Comparison of anti-anaerobic antimicrobial strategies in cancer patients with febrile neutropenia and gastrointestinal symptoms

**DOI:** 10.1186/1756-0500-7-614

**Published:** 2014-09-08

**Authors:** Regis G Rosa, Rodrigo P dos Santos, Luciano Z Goldani

**Affiliations:** Graduate Program in Medicine: Medical Sciences, Universidade Federal do Rio Grande do Sul, Porto Alegre, Brazil; Infection Control Committee of Hospital de Clínicas, Universidade Federal do Rio Grande do Sul, Porto Alegre, Brazil; Infectious Diseases Unit, Hospital de Clínicas de Porto Alegre, Ramiro Barcelos 2350, Room: 700, Porto Alegre, RS 90640-000 Brazil

**Keywords:** Bacteria, Anaerobic, Anti-bacterial agents, Febrile neutropenia, Immunocompromised host

## Abstract

**Background:**

The current study sought to compare 28-day mortality rates in cancer patients with febrile neutropenia (FN) and gastrointestinal (GI) symptoms who underwent monotherapy using an antibiotic with antipseudomonal and anti-anaerobic activity (piperacillin-tazobactam or a carbapenem) and a group treated with a combination of cefepime-metronidazole.

**Findings:**

We performed a prospective cohort study in a single tertiary hospital from October 2009 to August 2011. All consecutive adult cancer patients admitted with FN secondary to intensive chemotherapy and GI symptoms (abdominal pain, diarrhea or perianal pain) were evaluated. Kaplan-Meier curves were used for calculating time-dependent occurence of death. In total, 37 patients with FN and GI symptoms were evaluated (15 in monotherapy arm and 22 in the combination therapy arm). Treatment with combination cefepime and metronidazole resulted in a lower 28-day mortality rate compared with piperacillin-tazobactam or carbapenem monotherapy (0% versus 40%; log-rank *P*=0.002).

**Conclusions:**

Results of the present study suggest a significant reduction in mortality in cancer patients with FN and GI symptoms treated with combination cefepime-metronidazole therapy compared with monotherapy using agents with antipseudomonal and anti-anaerobic activity. Further randomized trials are warranted to confirm the superior results using combination therapy in patients with FN and GI symptoms.

## Findings

Febrile neutropenia (FN) induced by cytotoxic chemotherapy is known to predispose patients to anaerobic bacteremia [1]. Damage to the gastrointestinal (GI) mucosa caused by anti-cancer agents provides a portal of entry for commensal anaerobic bacteria. This fact, in association with a decreased host response to infection due to granulocytopenia and impaired cellular immunity, may contribute to bloodstream infection caused by bacterial translocation [2]. Despite its low incidence, anaerobic bacteremia is usually associated with high morbidity and mortality rates, especially in the context of cancer and immunosuppression [3–5].

Timely empirical therapy using an antipseudomonal, broad-spectrum antibiotic is part of the initial management of FN [6, 7]. Anti-anaerobic coverage is usually recommended by guidelines as part of the initial treatment when GI symptoms (eg, abdominal pain, diarrhea, perianal pain) are present [8, 9]. Unfortunately, only scarce data regarding the optimal anti-anaerobic antimicrobial strategy for patients with FN and GI symptoms are available. The aim of the present study was to compare the mortality rates of hospitalized adult cancer patients with FN and GI symptoms who either underwent monotherapy using an antibiotic with antipseudomonal and anti-anaerobic activity or were treated with a combination of cefepime and metronidazole.

### Study design and participants

A prospective cohort study was conducted in a single tertiary centre from October 2009 to August 2011. All consecutive cancer patients admitted to the hematology ward of the *Hospital de Clínicas de Porto Alegre* (Porto Alegre, Brazil) with neutropenia (absolute neutrophil count <500 cells/mm^3^, or <1000 cells/mm^3^ with an expectation of a decrease to <500 cells/mm^3^ during the ensuing 48 h), fever (a single axillary temperature measurement ≥38.5°C or sustained temperature ≥38.0°C over a 1 h period) and GI symptoms (abdominal pain, presence of loose or watery stool, or perianal pain) were eligible for the present study. Subjects who were receiving only palliative treatment or who had an indication for outpatient treatment or neutropenia due to a specific etiology other than an adverse reaction to chemotherapy were excluded. Patients were not allowed to reenter the study after a first episode of FN with GI symptoms.

### Definitions

The primary independent variable was the initial antimicrobial treatment administered by the medical care team. Due to observational study design, the research team did not influence treatment or diagnostic procedures. Patients were classified into two groups: subjects who received monotherapy using an antibiotic with antipseudomonal and anti-anaerobic activity (piperacillin-tazobactam 4.5 g intravenous [IV] over a 4 h period every 8 h, or imipenem-cilastatin 500 mg IV every 6 h or meropenem 1 g IV every 8 h); and subjects treated with a combination of cefepime 2 g IV every 8 h plus metronidazole 500 mg IV every 8 h. Clinical comorbidity was defined as the presence of heart failure, diabetes mellitus, chronic pulmonary disease or chronic liver disease. Nosocomial-acquired FN was defined as the onset of FN after 48 hours of hospitalization. High-dose chemotherapy was defined as induction chemotherapy or hematopoietic stem cell transplantation. The Multinational Association for Supportive Care in Cancer risk index score [10] was applied at the onset of fever to determine the risk for serious complications during an episode of FN; episodes were classified as high risk if the score was < 21 and low risk if the score was ≥ 21. Microbiological studies were performed at the onset of fever according to standards of practice and included two separate blood samples from two different anatomical sites for aerobic culture, and enzyme immunoassay testing for *Clostridium difficile* toxin A and B in a stool sample from patients with diarrhea. Antibiotic susceptibilities of the isolated pathogens were evaluated according to the recommendations of the Clinical and Laboratory Standards Institute [11]. Pseudomembranous colitis was diagnosed through a positive test for *C. difficile* toxin in a stool sample. Neutropenic enterocolitis was defined as bowel wall thickening >4 mm at the terminal ileus, cecum or ascending colon documented by abdominal computed tomography performed in patients who experienced abdominal pain.

### Outcome and follow-up

The primary outcome measure of the present study was mortality 28 days after the onset of FN. Patients were followed-up through interviews and medical record reviews using a standardized data collection instrument by researchers who were not associated with the assistant physician’s team. Follow-up was maintained for 28 days after the onset of fever in neutropenic patients. For subjects who were discharged before 28 days, follow-up telephone calls were made on the 28th day after the onset of FN to determine whether they remained alive.

### Statistical analysis

The *χ*^2^ and Fisher’s exact tests were used to compare categorical variables; the Mann–Whitney *U* test was used to compare continuous variables. Kaplan-Meier curves were used to calculate the time-dependent occurrence of death. The log-rank test was used for comparisons between groups. Statistical analysis was performed using STATA version 12 (Stata Corp LP, USA).

### Ethics issues

The Institutional Review Board of the *Hospital de Clínicas de Porto Alegre* approved the study and written informed consent was obtained from all study participants.

### Results

In total, 37 patients were evaluated during the study period; patients with hematological malignancies comprised 75% of the study population. The predominant neoplastic diseases were acute myeloid leukemia (35.1%), lymphoma (21.6%), acute lymphoblastic leukemia (16.2%) and multiple myeloma (16.2%). The proportion of patients who underwent high-dose chemotherapy was 56.7%. Antibiotic prophylaxis with fluoroquinolones was not administered to any patient. Fifteen subjects initially underwent monotherapy using an antibiotic with antipseudomonal and anti-anaerobic activity (12 received piperacillin-tazobactam; three received a carbapenem), while 22 subjects were initially treated with a combination of cefepime and metronidazole. The overall mortality rate in the present cohort was 16.2% (six patients).

Characteristics of the patients who underwent monotherapy using an antibiotic with antipseudomonal and anti-anaerobic activity and those treated with a combination of cefepime and metronidazole are presented in Table 1. There were no statistically significant differences between the two groups with regard to baseline characteristics. The incidence of neutropenic enterocolitis, pseudomembranous colitis, aerobic bacteremia and *in vitro* resistance of aerobic blood isolates to initial antibiotic treatment was also similar for the two study arms.

**Table 1:** **Characteristics of patients with febrile neutropenia (FN) and gastrointestinal symptoms**

Variable	Combination therapy ^a^group (n=22)	Monotherapy ^b^group (n=15)	***P***
Age, years, mean (SD)	44.7 (14.3)	41.2 (11.0)	0.43
Female sex	15 (68.1)	5 (33.3)	0.05
Type of Cancer			0.18^c^
Acute myeloid leukemia	6 (27.3)	7 (46.7)	
Acute lymphoblastic leukemia	4 (18.2)	2 (13.3)	
Chronic myeloid leukemia	1 (4.5)	2 (13.3)	
Multiple myeloma	6 (27.3)	0 (0)	
Lymphoma	5 (22.7)	3 (20.0)	
Other solid tumors	0 (0)	1 (6.7)	
Relapsing underlying disease	13 (59.0)	12 (80.0)	0.28
Clinical comorbidity	5 (22.7)	7 (46.6)	0.16
High dose chemotherapy regimens	12 (54.5)	9 (60.0)	0.74
Nosocomial-acquired episode of FN	19 (86.3)	13 (86.6)	0.97
ANC at the time of diagnosis of FN, median cells/mm^3^ (IQR)	80 (160)	130 (360)	0.47
ANC <100 cells/mm^3^ at the time of diagnosis of FN	13 (59.0)	6 (40.0)	0.32
High-risk MASCC score	8 (36.3)	2 (13.3)	0.15
Neutropenic enterocolitis	1 (4.5)	1 (6.6)	0.99
Pseudomembranous colitis	1 (4.5)	1 (6.6)	0.99
Documented aerobic bacteremia	8 (36.3)	9 (60.0)	0.19
*In vitro* resistance of aerobic blood isolates to initial antibiotic treatment	1 (4.5)	2 (13.3)	0.55

Data presented as n (%) unless otherwise indicated. ^a^Cefepime + metronidazole; ^b^Piperacillin-tazobactam or imipenem or meropenem; ^c^Chi-square test for goodness of fit; ANC Absolute neutrophil count; HSCT Hematopoietic stem cell transplantation; IQR Interquartile range (P75–P25); MASCC Multinational Association for Supportive Care in Cancer; SD Standard deviation.

**Figure 1:**
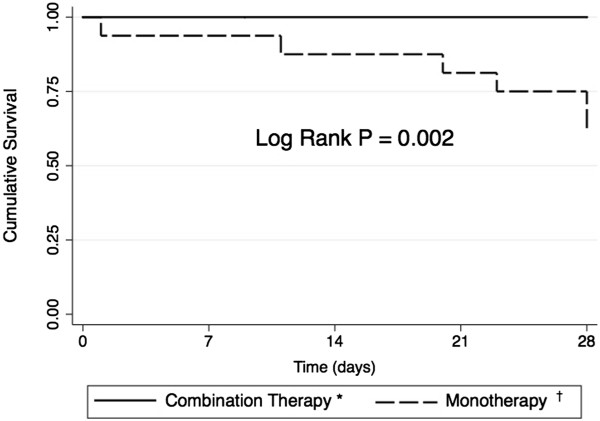
**Survival curves according to antibiotic strategy in patients with febrile neutropenia and gastrointestinal symptoms.** Figure legend: *Combination therapy: cefepime + metronidazole; †monotherapy: piperacillin-tazobactam or imipenem or meropenem.

The 28-day mortality rate was significantly lower in the combination therapy group compared with the monotherapy group (0% versus 40.0%; log rank *P*=0.002) (Figure 1). The assessment of whether mortality was attributable to infection was concordant in all six patients who died.

### Conclusions

The present study demonstrated higher survival rates in adult cancer patients with FN who presented with GI symptoms treated with a combination of a fourth-generation cephalosporin plus metronidazole compared with those who underwent monotherapy using a broad-spectrum antibiotic with antipseudomonal and anti-anaerobic activity.

The findings of this study have scientific plausibility given that an antimicrobial strategy using a combination of cefepime and metronidazole has better coverage for anaerobic pathogens than a monotherapy strategy (piperacillin-tazobactan or a carbapenem). This hypothesis is supported by the fact that, in our study, the incidence of aerobic bacteremia and the proportion of *in vitro* resistance of aerobic blood isolates to initial antibiotic treatment were similar in the two treatment arms. Moreover, previous efficacy studies involving distinct populations have reported results convergent with those obtained in the present cohort. In a randomized clinical trial involving 122 patients with intra-abdominal infections, Garbino et al. [12] reported higher success rates with combination cefepime-metronidazole treatment compared with imipenem-cilastatin monotherapy (87% versus 72%; *P*=0.004). Similarly, Barie et al. [13] reported higher cure rates for combination treatment with cefepime plus metronidazole compared with imipenem-cilastatin treatment in adult subjects with complicated intra-abdominal infections (88% versus 76%; *P*=0.02). The high impact in risk reduction of mortality found in our study using combination cefepime-metronidazole treatment may be due the high levels of characteristically expected morbidity and mortality of our study population, which consisted of a large proportion of high-risk neutropenic patients in whom the correct choice of antimicrobial strategy is of paramount importance. In addition, considering the low incidence of *C. difficile* colitis in both arms of our study, empirical addition of metronidazole to the initial regimen suggests improved efficacy against anaerobes other than *C. difficile* in this setting. In fact, metronidazole has good activity against pathogenic anaerobic bacteria, and a favourable pharmacokinetic and pharmacodynamic profile [14]; furthermore, combining metronidazole with cefepime in patients with FN and GI symptoms may provide additional advantages over monotherapy strategies using piperacillin-tazobactam or carbapenems because this combination is less expensive and has a lower potential of inducing antimicrobial resistance, especially in Gram-negative bacteria.

This study has some limitations, mainly related to the small study population and to the unicentre observational design; however, proper measurement of variables and outcomes with previously defined objective criteria, use of standardized data collection, as well as follow-up for research team that was not related to care, minimized the possibility of systematic errors.

Given the scarce data in the literature regarding this important issue, larger randomized trials are required to confirm the superiority of combination cefepime-metronidazole treatment over broad-spectrum monotherapies in patients with FN and GI symptoms.

## Abbreviations

FN: Febrile Neutropaenia
GI: Gastrointestinal
ANC: Absolute Neutrophill Count
MASCC: The Multinational Association for Supportive Care in Cancer.
